# Establishment of patient-derived xenografts for neuroendocrine tumors in the avian embryo model

**DOI:** 10.1530/ERC-25-0377

**Published:** 2026-03-23

**Authors:** Nilakshi Kulathunga, Rusha Chakraborty, Yan Li, Emmanuel Cherin, Ziyi Zoey Wang, Sara Mar, Tina Khazaee, Alexandra Misura, Alberto Lens-Pardo, Po Hien Ear, Jörg Schrader, Rocio Garcia-Carbonero, Lina Chen, Weei-Yuarn Huang, Hubert Tsui, Simron Singh, Calvin Law, Julie Hallet, Christine E Demore, Hon S Leong, Iacovos P Michael

**Affiliations:** ^1^ Biological Sciences Platform, Sunnybrook Research Institute, Toronto, Ontario, Canada; ^2^ Physical Sciences Platform, Sunnybrook Research Institute, Toronto, Ontario, Canada; ^3^ Department of Medical Biophysics, University of Toronto, Toronto, Ontario, Canada; ^4^ Sunnybrook Biobank, Department of Laboratory Medicine and Molecular Diagnostics, Precision Diagnostics and Therapeutics Program, Sunnybrook Health Sciences Centre, Toronto, Ontario, Canada; ^5^ Oncology Department, Hospital Universitario 12 de Octubre, Centro de Oncologia Experimental, Instituto de Investigacion Sanitaria Hospital 12 de Octubre (imas12), Facultad de Medicina, Universidad Complutense de Madrid (UCM), CNIO, Madrid, Spain; ^6^ Department of Surgery, University of Iowa Carver College of Medicine, Iowa City, Iowa, USA; ^7^ First Department of Medicine, University Hospital Hamburg-Eppendorf, Hamburg, Germany; ^8^ Department of Laboratory Medicine and Pathobiology, University of Toronto, Toronto, Ontario, Canada; ^9^ Precision Diagnostics and Therapeutics Program, Division of Anatomic Pathology, Department of Laboratory Medicine and Molecular Diagnostics, Sunnybrook Health Sciences Centre, Toronto, Ontario, Canada; ^10^ Department of Immunology, Temerty Faculty of Medicine, University of Toronto, Toronto, Canada; ^11^ Odette Cancer Centre, Sunnybrook Health Sciences Centre, Toronto, Ontario, Canada; ^12^ Department of Medicine, University of Toronto, Toronto, Ontario, Canada; ^13^ Department of Surgery, University of Toronto, Toronto, Ontario, Canada

**Keywords:** neuroendocrine tumors, patient-derived xenografts, avian *ex ovo* model, preclinical models

## Abstract

Elucidating the mechanisms underlying cancer progression and identifying tailored therapies for patients can be enhanced by using patient-derived preclinical models. In this study, we investigated whether patient-derived xenograft (PDX) models of well-differentiated neuroendocrine tumors (NETs) could be established in the avian embryo *ex ovo* model. We found that fresh surgical tumor samples from well-differentiated primary small-intestine and pancreatic NETs, as well as metastatic sites, exhibited engraftment rates exceeding 80% in the avian embryo model. The NET PDXs in the avian model preserved the distinct histological features of NETs, including characteristic tumor nests and the ‘salt and pepper’ chromatin pattern in nuclei. Using immunostaining, we showed that the engrafted patient tumor fragments remained viable and maintained the proliferation rate, e.g. tumor grade, of the corresponding patient tumors. The NET PDXs continued to express characteristic neuroendocrine markers, such as the insulinoma-associated 1 (INSM1) transcription factor and chromogranin A (CgA). Importantly, they also retained the patient’s somatostatin receptor 2 (SSTR2) expression pattern in cancer cells, which is the target of radioligand therapy. Using high-frequency ultrasound imaging and immunostaining, we also demonstrated that the engrafted tumor specimens were vascularized and exhibited functional blood perfusion. Overall, this is the first study to demonstrate the feasibility and characterize PDXs of well-differentiated NETs in the avian embryo model.

## Introduction

Gastroenteropancreatic neuroendocrine neoplasms (GEP-NENs) arise from the malignant transformation of neuroendocrine cells in the pancreas, stomach, and small and large intestines. According to the current World Health Organization classification, neuroendocrine neoplasms are histologically categorized into well-differentiated neuroendocrine tumors (NETs) and poorly differentiated neuroendocrine cancers (NECs) (1). NETs are further classified, based on their proliferation and mitotic index, into three grades: grade 1 (Ki-67 < 2%), grade 2 (Ki-67 2–20%), and grade 3 (Ki-67 > 20%). NECs are all considered high-grade cancers with a Ki-67 index greater than 50%. While the incidence of GEP-NENs is increasing worldwide (2, 3, 4), there is an unmet need to understand the underlying molecular pathways and develop patient-specific therapies (5).

Cancer models are crucial for understanding the molecular pathways involved during tumor progression and for identifying new therapies for specific tumor molecular subtypes. For example, using mouse orthotopic and transgenic models, we showed that miRNAs regulate phenotypic plasticity, invasion, and metastasis of pancreatic neuroendocrine tumors (PanNETs) (6, 7). In addition, we unveiled that antiangiogenic therapy enhances the efficacy of immune checkpoint inhibitors for PanNETs (8). Transgenic mouse models also guided clinical trials and the approval of therapies for neuroendocrine tumors, such as antiangiogenic therapy (9, 10). Patient-derived preclinical models are cancer models that use patient tumor specimens to enable precision oncology, for example, by assessing responses to various drug treatments and combinations. Recently, several studies have reported the establishment of human patient-derived preclinical models for NENs, including patient-derived tumor organoids, tumoroids, and xenografts in immunocompromised mice (11, 12, 13, 14, 15, 16, 17).

In this study, we examined whether the chorioallantoic membrane (CAM) of the avian embryo can be used for the engraftment of patient-derived tumor fragments from patients with well-differentiated NETs. The avian CAM is a highly vascularized membrane that surrounds the entire avian embryo and serves as the respiratory organ (18). The high vascularity of the CAM, combined with its immunodeficient nature up until term, is highly permissive of engraftment of cells and tissues from xenogeneic species. The CAM model has been used to study the response to various treatments, such as chemotherapy and radiation therapy, as well as to study mechanisms involved in cancer progression, including metastasis (19, 20, 21, 22, 23, 24). In addition, it offers the advantage that it is amenable to imaging, such as PET, ultrasound (25, 26), fluorescence (27, 28, 29, 30, 31), and bioluminescence (20, 32, 33). Finally, the avian CAM model is cost-effective and provides an alternative *in vivo* vascularized model for modeling PDXs. Previous studies have used the CAM assay to engraft cell lines from neuroendocrine neoplasms, including Merkel cell carcinoma and high-grade PanNETs (34, 35). In this study, we demonstrated that patient-derived tumor samples from patients with low-grade, well-differentiated NETs can successfully be engrafted in the avian embryo model.

## Materials and methods

### GEP-NET cell lines

The NT-18 LM cell line was obtained from the Jörg Schrader group at the University Medical Center Hamburg-Eppendorf, Germany (36), and cultured in RPMI (350-000 CL; WISENT, Canada) supplemented with 10% fetal bovine serum (FBS; Gibco, USA), 20 ng/mL human EGF (AF-100-15; PeproTech, USA), 10 ng/mL basic FGF (AF-100-18B; PeproTech, USA), and 200 μM GlutaMAX (35050061; Gibco, USA), on collagen-coated (CAS 9007-34-5; C7521; Sigma, Germany) cell culture plates. The BON-1 cell line was obtained from the Rocio Garcia-Carbonero’s group at Hospital Universitario Doce de Octubre in Spain and cultured in DMEM (319-005 CL; WISENT, CN) with 10% FBS (Gibco, USA). The NEC913 and NEC1452 cell lines were obtained from the Po Hien Ear group at the University of Iowa (37) and cultured in DMEM/F12 (319-075 CL; WISENT, CN) medium supplemented with 10% FBS, 1% penicillin/streptomycin, 1% L-glutamine, insulin, and nicotinamide. All cell lines were tested negative for mycoplasma and were maintained at 37°C in a 5% CO_2_ incubator. Cell line identity was verified by short tandem repeat profiling performed at The Centre for Applied Genomics (TCAG), The Hospital for Sick Children (SickKids), Toronto, Canada. No evidence of cross-contamination or misidentification was detected (Supplementary Table 1 (see section on Supplementary materials given at the end of the article)).

### GEP-NET human samples

Candidate GEP-NET patients were identified from the Susan Leslie Clinic for Neuroendocrine Tumors at the Odette Cancer Centre at Sunnybrook Health Sciences Centre. Following written informed consent through the Sunnybrook Biobank – Neuroendocrine Neoplasms, fresh surplus surgical tissue specimens in DMEM/F12 + 1% penicillin/streptomycin were diverted for the development of PDX models following initial clinical grossing by the staff of the Department of Laboratory Medicine and Molecular Diagnostics (LMMD) pathology staff. Research tissue adjacent to clinically sampled sites was prioritized where feasible. Tumor samples and associated histological slides were coded with anonymized identifiers, and all experiments adhered to accepted ethical guidelines. The study was approved by the Sunnybrook Health Sciences Centre’s Research Ethics Committee (Protocol # 5514).

### Establishment of PDXs on the chorioallantoic membrane of avian embryos

Fertilized duck eggs (White Pekin ducks; King Cole Farm, Canada) were incubated in a rotating, humidified (40% relative humidity) egg incubator (Maru Deluxe, USA) for three days at 37.5°C. At embryonic day 4, ED04, the eggshell was opened, and the avian embryo, along with its chorioallantoic membrane (CAM), was transferred to a sterile plastic boat covered with a lid (*ex ovo* method) and further incubated at 37.5°C, in a sterile environment in a separate incubator (Caron Scientific, USA) (27, 30, 38, 39). Cells or fresh human tissue implantation was performed at ED08. NEN cancer cells were detached using TrypLE^TM^ (Thermo Fisher Scientific, USA), neutralized, and washed three times with PBS. The cells were then pelleted and reconstituted in a 1:1 dilution of growth factor-reduced Geltrex (A1413201; Thermo Fisher Scientific, USA). A cell pellet of ten microliters containing 5 × 10^6^ cells was implanted into the CAM after scoring the tissue using blotting paper. Surgical tumor fragments were cut into 3–5 mm^3^ pieces and implanted on the CAM after scoring the surface of the CAM tissue. Fifteen microliters of Geltrex were added on top of the tumor fragment. At the endpoint (ED16 to ED18), tumors were visualized using bright-field and high-frequency ultrasound imaging to assess tumor volume and blood flow. Finally, the tumors were resected and fixed for histological analysis.

### Bright-field imaging

Avian embryos were visualized to confirm tumor formation, using a Leica stereomicroscope equipped with a digital camera (Leica, Germany). Images were captured using NIS Elements (version 5.21.02) Imaging Software, Nikon Instruments Inc.

### High-frequency ultrasound (HF-US) imaging and volume quantification

Ultrasound measurements were performed using a Vevo 2,100 high-frequency imaging system (FujiFilm-Visualsonics, Canada), equipped with a 50 MHz linear array transducer (MS-700). Acquired volumetric and high-frequency (>100 Hz) B-mode data were analyzed using an image analysis pipeline specifically developed to estimate CAM tumor volume and blood flow. The margins of the tumors were manually delineated in all the 2D imaging planes acquired across the sample (step size: 0.51 mm), and the volume was calculated using VevoLab (FujiFilm-Visualsonics, Canada).

### Tumor blood flow quantification

Blood flow analysis was performed as recently described (40). In brief, the image analysis entails three steps: i) removal of any motion artifacts in acquired image datasets, ii) removal of any non-tumor signal so that calculations of tumor volume can be automatically generated, and iii) quantitation of tumor perfusion by estimation of the mean speckle variance of the signal from red blood cells circulating in its microvasculature within the imaging plane.

### Lectin injection

To label the CAM vasculature, 0.05 mg of biotinylated lens culinaris agglutinin (LCA) (B-1045-5; Vector Laboratories, USA) diluted in 100 μL of PBS (sterile-filtered) were intravenously injected into a small vein of the CAM using a glass needle 20 min prior to tumor harvesting. The harvested tumors were then subjected to histological analysis (41).

### Histology

At the endpoint, PDXs were harvested and fixed in 4% zinc formalin (Sigma-Aldrich Z2902) at 4°C overnight. After the serial dehydration process, tumor tissue samples were embedded in paraffin. Formalin-fixed paraffin-embedded (FFPE) samples were cross-sectioned (4 μm) using a microtome. Deparaffinization was performed in xylene, and rehydration was achieved over an ethanol gradient. Hematoxylin and eosin (H&E) staining was done using an automated stainer (ST5010 Autostainer XL; Leica Biosystems, Canada). The histology of PDXs was examined by clinical pathologists (LC and WYH) at the Sunnybrook Health Sciences Centre. Immunohistochemistry analysis was performed on deparaffinized tissue sections after antigen retrieval using sodium citrate (10 mM-pH 6.0) in a decloaking chamber (Biocare Medical, USA). The primary antibodies against the following antigens were used: chromogranin A (CgA, 1:500, NBP2-32956; Novus, USA), somatostatin receptor 2 (SSTR2; 1:500, ab134152; Abcam, UK), Ki-67 (1:1,000; 12075; Cell Signaling Technology, USA), cleaved caspase 3 (CC-3; 1:250, 9661; Cell Signalling Technology, USA), and INSM1 (1:250; sc-271408; Santa Cruz Biotechnology, USA). Conjugated secondary antibodies used were Goat anti-Mouse Alexa Fluor™ 647 (A-21235), Goat anti-Mouse Alexa Fluor™ 750 (A-2103), Goat anti-Rabbit Alexa Fluor™ 647 (A-21244), and Goat anti-Rabbit Alexa Fluor™ 750 (A-21039) at 1:1,000 dilution (Invitrogen, Thermo Fisher Scientific, USA). Stained slides were visualized, and images were acquired using Huron TissueScope LE (H&E) and Zeiss AxioObserver 3/5/7 KMAT (H&E and stained slides) and analyzed using Fiji-ImageJ. H&E and Ki-67 immunostained slides of the patient’s surgically resected tumor samples were obtained from the hospital’s pathology department.

## Results

### The avian embryo *ex ovo* model enables the engraftment of patient-derived neuroendocrine tumor fragments

**Figure 1:**
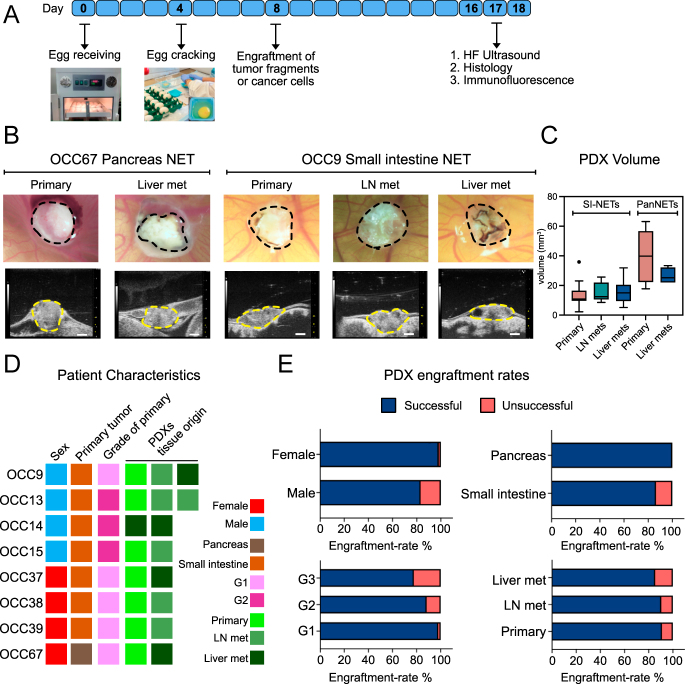
Establishment of human NET patient-derived xenografts (PDXs) within the avian embryo *ex ovo* model. (A) Flow chart of the PDX establishment procedure in the avian embryo *ex ovo* model. The numbers represent embryonic day of development. (B) Representative PDXs from PanNET and SI-NET primary tumors, lymph node, and liver metastasis. Top row: bright-field images. Bottom row: ultrasound B-mode images. Scale bar: 1,000 μm. (C) PDX tumor volume at the experimental endpoint, measured by ultrasound imaging. (D) Patient and NET characteristics from which tumor fragments were used to establish PDXs. PDXs were established from various tissue specimens from each patient, as indicated by the ‘tissue origin’. (E) PDX engraftment rate according to the patient’s sex, primary tumor location, grade, and tissue of origin. A full color version of this figure is available at https://doi.org/10.1530/ERC-25-0377.

In this study, we examined whether we could develop patient-derived NEN models using the avian *ex ovo* model. In this model, fertilized duck eggs are inoculated at 37.5°C for 4 days before the shell is opened and the avian embryo, along with the chorioallantoic membrane (CAM), is transferred to a square petri dish at embryonic day 4 (ED04). At ED08, the specimens are engrafted onto the CAM of fertilized duck eggs (Fig. 1A). To examine the engraftment capabilities of NENs in the avian model, we utilized the PanNEN cell lines NT-18LM and BON-1, as well as the SI-NEN cell lines NEC913 and NEC1452. A total of 5 × 10^6^ cells from each cell line were suspended in 10 μL of Geltrex and implanted on the CAM of the avian egg. We observed the establishment of solid tumors from all cell lines at the implantation site using bright-field microscopy and ultrasound imaging (Supplementary Fig. 1A and 1B). All NEN cell lines formed tumors in the avian model, with an approximate tumor formation success rate of over 90% (data not shown). To further characterize the NEN cell line-derived *ex ovo* tumors, we harvested the tumors at ED18 for histological analysis using hematoxylin and eosin (H&E) and immunohistochemical (IHC) staining. H&E staining of formalin-fixed paraffin-embedded sections confirmed that the tumors derived from the NEN cell lines preserved the unique morphological feature of granulated ‘salt and pepper’ chromatin appearance (Supplementary Fig. 1C).

Having established that the avian embryo *ex ovo* model is suitable for engrafting human NEN cell lines, we next examined whether we could successfully engraft fresh surgical tumor fragments of well-differentiated NETs. The human tumor specimens used in this study were derived from surplus surgical samples taken from patients undergoing routine surgical debulking at the Odette Cancer Centre at the Sunnybrook Health Sciences Centre. To facilitate the establishment of NET *ex ovo* models, we collaborated with the surgical oncology team and the pathology department to ensure that patient samples were directed to our laboratory fresh in media within two hours post-surgery. After surgery, samples were processed in the anatomy laboratory; a tissue fragment was kept for accurate diagnosis, while the remainder of the sample was used for engraftment. The fresh tumor samples were dissected into approximately 3–5 mm^3^ fragments and transplanted onto the CAM at ED08, followed by the application of Geltrex on top of the fragment. From each patient sample, we generated 8–12 individual PDXs in the avian model, depending on the sample size.

Patient-derived tumor tissues engrafted in the avian *ex ovo* model were imaged using bright-field imaging (Fig. 1B), and their volume at the endpoint of the assay, at ED16–ED18, was measured using high-frequency ultrasound (HF-US) (Fig. 1B and C). Successful engraftments were determined by the preservation of the patient tumor fragment at the endpoint, as assessed by bright-field imaging and immunostaining (see below). Overall, we were able to derive PDXs in the avian embryo from well-differentiated NETs originating from various tissues, including small-intestinal and pancreatic primary NETs, lymph node and liver metastases, and different grades (Fig. 1D and E and Supplementary Table 2).

### PDXs within the avian embryo maintain neuroendocrine histological features

**Figure 2:**
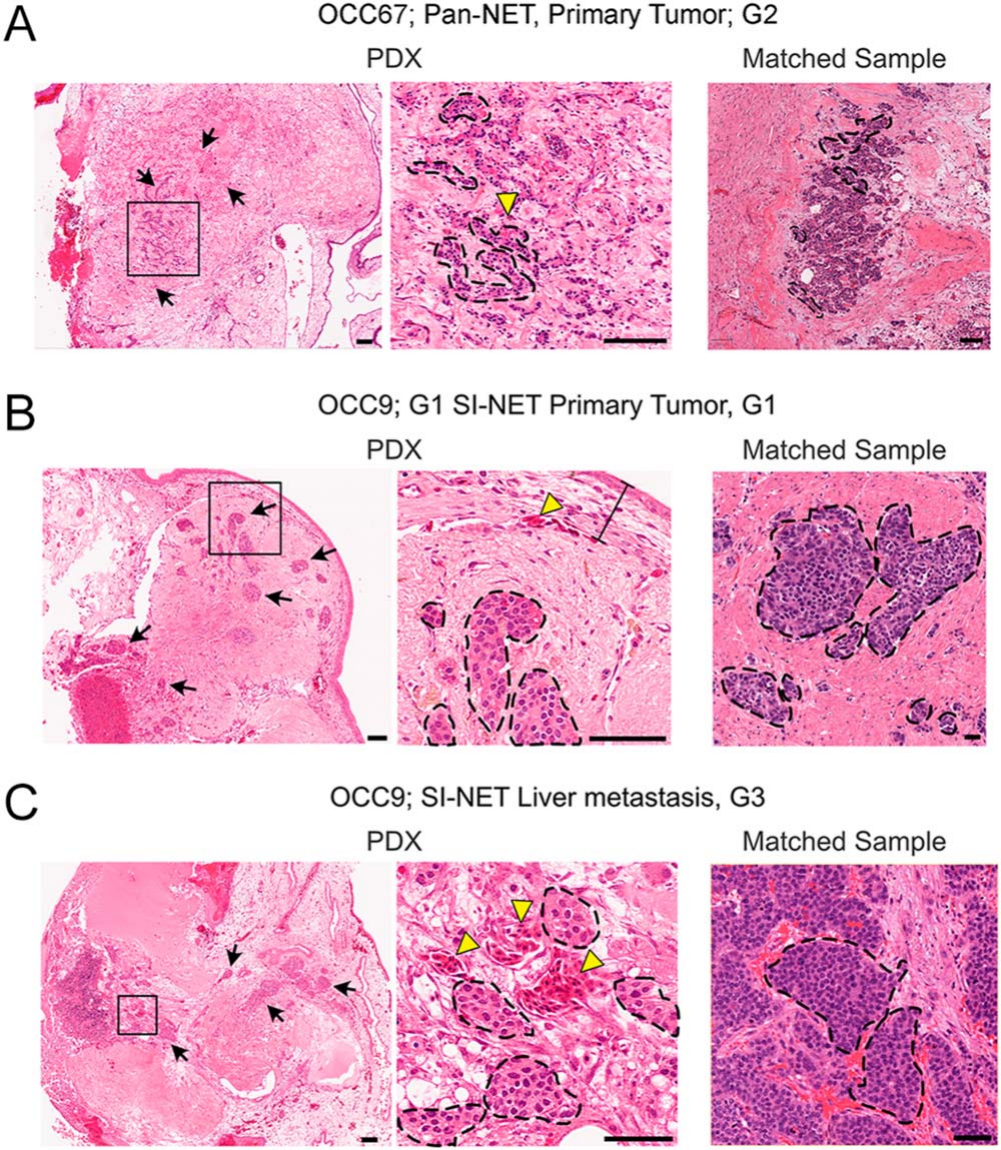
Histological characterization of NET PDXs within the avian embryo. (A, B, C) Representative hematoxylin and eosin (H&E) images of PDXs derived from PanNETs (A; OCC67) and SI-NETs (B and C; OCC9). PDXs from matched primary and liver metastasis are shown for OCC9 (B and C). Corresponding H&E staining from original tumor samples is shown in the right image of each panel. The black closed dashed lines and black arrows; neuroendocrine tumor cell clusters. Yellow arrowheads: avian red blood cells. The black line in panel B demarcates the avian chorioallantoic membrane. Scale bar: 100 μm. A full color version of this figure is available at https://doi.org/10.1530/ERC-25-0377.

To determine whether the tumors grown in the CAM of the avian embryo retain the characteristic morphological features of low-grade, well-differentiated neuroendocrine tumors, we performed H&E staining on several samples from different tissue origins and tumor grades. The samples were reviewed by the NEN pathologists at our institute and compared with the corresponding patient samples. We observed the preservation of characteristic tumor nests in the PDX tumors and the ‘salt and pepper’ chromatin pattern in nuclei (Fig. 2). Tumor nests were identified in PDX samples across all tissue sites, including PanNETs, SI-NETs, and both primary and metastatic tumors.

### PDXs within the avian embryo retain the proliferation rate of the patient’s tumor and exhibit low levels of apoptosis

To further characterize the PDXs in the avian embryo *ex ovo* model, we immunostained a subset of samples with the neuroendocrine markers, such as insulinoma-associated 1 (INSM1), chromogranin A (CgA), and synaptophysin (SYN), along with the proliferation and apoptosis markers Ki-67 and cleaved caspase 3 (CC3), respectively. The expression of INSM1 and SYN was homogeneous in the NT-18, NEC1452, and NEC913 xenografts, whereas CgA either was expressed at lower levels (NT-18 and NEC1452) or showed heterogeneous expression (NEC913) (Supplementary Fig. 2). We observed that the BON-1 cell line had a heterogeneous expression of all three neuroendocrine markers, having predominantly two cancer cell populations, INSM1^high^; CgA^high^; SYN^high^ and INSM1^low^; CgA^low^; SYN^low^ (Supplementary Fig. 2). Heterogeneous expression of these markers has been previously reported in human tissue microarrays from pancreatic NENs (42). The human cell-derived *ex ovo* models exhibited high proliferation and low apoptosis, indicating that the avian model supports the growth of neuroendocrine cancer cells (Supplementary Fig. 3).

**Figure 3:**
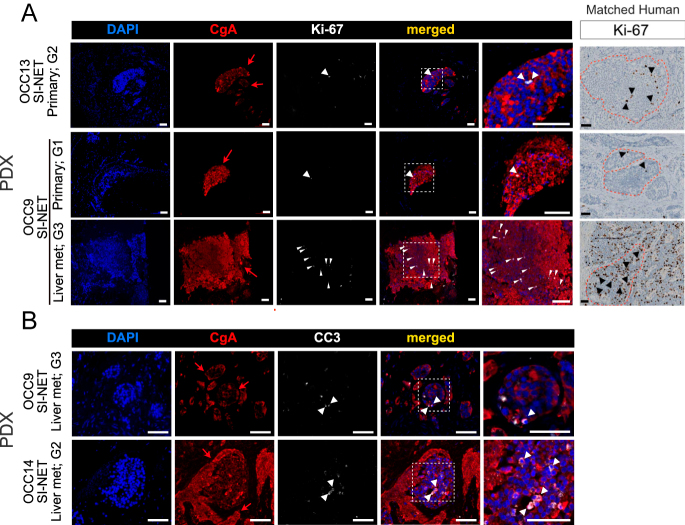
Characterization of proliferation and apoptosis of NET PDXs within the avian embryo. (A) Representative images of CgA (neuroendocrine cancer cell marker) and Ki-67 (proliferation marker) immunostaining. Corresponding Ki-67 staining from human samples is shown in the right image of each panel. Red arrows: NET clusters. White and black arrowheads: proliferating cells. Scale bar: 100 μm. (B) Representative images of CgA (neuroendocrine cancer cell marker) and cleaved caspase 3 (CC3; apoptosis marker) immunostaining. Red arrows: NET clusters. White arrowheads: apoptotic cells. Scale bar: 100 μm. A full color version of this figure is available at https://doi.org/10.1530/ERC-25-0377.

PDXs derived from G1 and G2 SI-NET patient samples, such as OCC9 and OCC13, respectively, maintained a lower proliferation rate, with only a few Ki-67-positive cells per tumor nest (Fig. 3A). Notably, the PDXs established from the G3 liver metastasis of patient OCC9 exhibited a higher proliferation rate than those from the matched G2 primary tumor, consistent with the patient samples (Fig. 3A). In all cases, we observed a low apoptosis rate, further indicating that the avian embryo model is a suitable host for xenografting samples from neuroendocrine tumors (Fig. 3B). Overall, these *in vivo* findings suggest that NET PDXs maintain their original tissue histological characteristics.

### SSTR2 expression is maintained in NET PDXs within the avian embryo

Somatostatin analogs and radioligand therapy, in the form of a radionuclide conjugated to the somatostatin analog, are two of the main therapies used in the clinic for NENs. Therefore, we also examined whether the tumors retain SSTR2 expression. First, we characterize SSTR2 expression in cell line-derived *ex ovo* tumors. In accordance with previous studies, we observed low SSTR2 expression in the NT-18LM tumors, whereas the BON-1 tumors showed heterogeneous SSTR2 expression (Supplementary Fig. 4). Heterogeneous SSTR2 expression has also been reported *in vivo* in BON-1-derived mouse tumors (43). In the case of the SI-NEN-derived tumors, we observed high SSTR2 expression in the NEC913 tumors and expression in only a few cells in the NEC1452 tumors (Supplementary Fig. 4), as previously reported (37).

**Figure 4:**
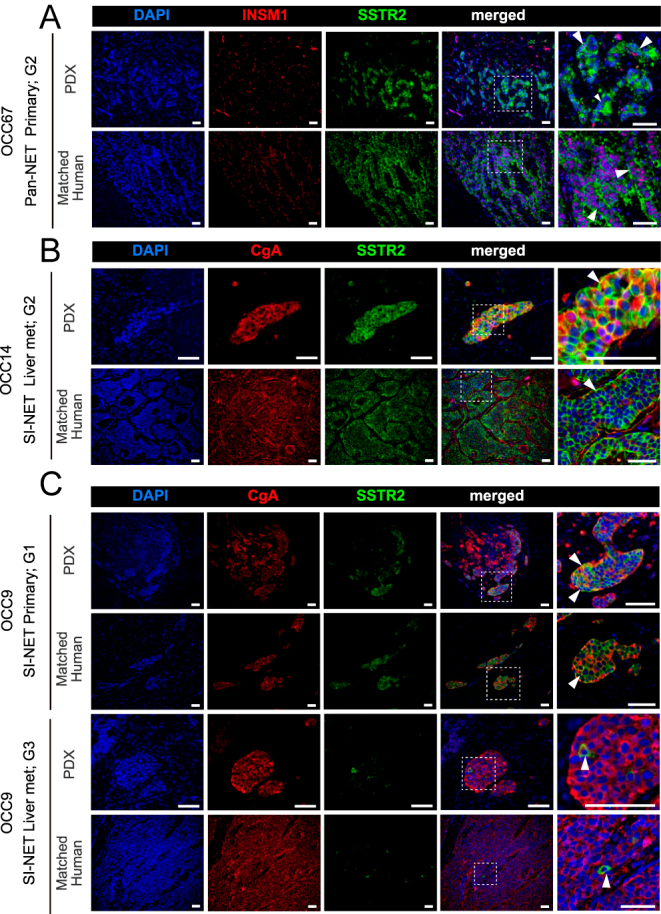
Characterization of somatostatin receptor 2 (SSTR2) expression in NET PDXs within the avian embryo. (A, B, C) Representative images of SSTR2 immunostaining along with either INSM1 or CgA (neuroendocrine cancer cell markers). PDXs from matched primary and liver metastasis are shown for OCC9 (C). Corresponding staining from original tumor samples is shown in the bottom row of each panel. White arrowheads: membrane SSTR2 expression in NET cells. Scale bar: 50 μm. A full color version of this figure is available at https://doi.org/10.1530/ERC-25-0377.

We then characterized SSTR2 expression in the PDXs. We observed that PDXs established from low-grade, well-differentiated NETs maintain high expression of SSTR2 in the cell membrane of cancer cells, similar to the corresponding clinical samples (Fig. 4A, B, C and Supplementary Fig. 5). Notably, in the matched primary and liver metastasis samples from patient OCC9, we observed a loss of SSTR2 expression in the liver metastasis samples compared to the primary ones, which was consistent in both the PDXs and patient samples (Fig. 4C). Overall, these results indicate that PDXs established in the avian embryo maintain the patient-specific pattern of SSTR2 expression.

### NET PDXs within the avian embryo are vascularized by functional vessels

**Figure 5:**
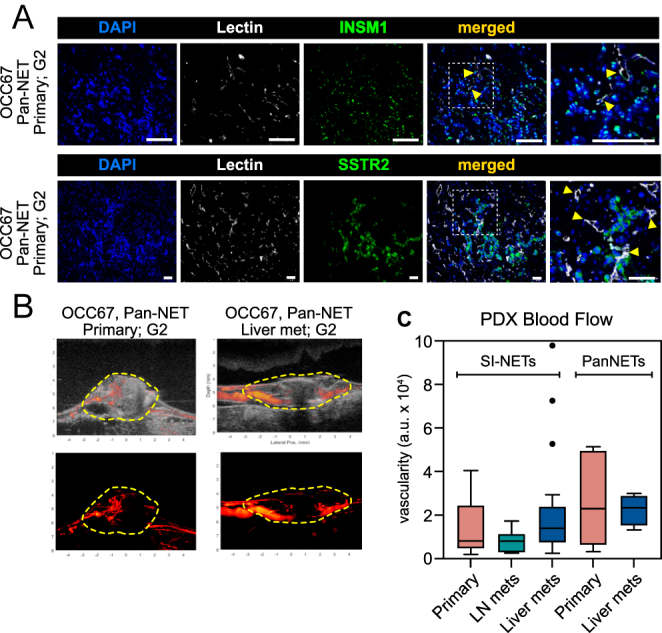
Characterization of vascular network and blood perfusion of NET PDXs within the avian embryo. (A) Representative images of lectin labeling of endothelial cells in NET PDXs as a marker of blood perfusion of the tumor. Lectin was injected into the avian CAM blood circulation prior to PDX harvesting. INSM1 and SSTR2 immunostaining mark NET cancer cells. Yellow arrowheads: endothelial cells. Scale bar: 100 μm. (B) Representative images of high-frequency ultrasound B-mode and speckle variance imaging (blood perfusion) of NET PDXs. Yellow closed dashed lines: neuroendocrine tumor xenograft. (C) Quantification of PDXs perfusion using speckle variance imaging. A full color version of this figure is available at https://doi.org/10.1530/ERC-25-0377.

One of the main advantages of the avian embryo model is its rich vascular network, which can support cell lines and tumor grafts by supplying nutrients via the blood supply. In our histology images, we observed the presence of avian red blood cells, characterized by their oval shape and the presence of a nucleus, adjacent to cancer cells, indicating that NEN PDXs in the avian model are vascularized (Fig. 2 and Supplementary Fig. 1). To confirm the presence of vessels in tumors, we injected *Lycopersicon esculentum* lectin, which binds to the glycocalyx of endothelial cells of the CAM vessels adjacent to the PDX before tumor dissection. Using immunostaining, we detected vessels in the PDXs adjacent to the cancer cells (Fig. 5A).

To further confirm the functionality of the blood vessels in the PDX models, we used our image analysis pipeline to quantify blood flow in the avian embryo *ex ovo* model (40). We observed varying levels of blood circulation in all PDXs, characterized by both large vessels, usually located at the periphery of the PDXs, along with additional smaller vessels within the PDXs (Fig. 5B and C and Supplementary Videos 1 and 2). In conclusion, our findings suggest that NET PDXs in the avian embryo model have a functional vascular network.

## Discussion

Over the past decade, efforts from various groups have led to the establishment of new models for neuroendocrine neoplasms, including human cell lines (36, 44), along with models using patient-derived samples, such as tumor slices (45), organoids (12, 14, 46), tumoroids (13, 15), and cell implantation in the zebrafish model (47). Our study demonstrated that the avian embryo *ex ovo* model is a reliable and viable platform for engrafting tumor fragments from low-grade, well-differentiated neuroendocrine tumors, thereby establishing a new preclinical model for NET PDXs.

The avian embryo model involves engrafting patient-derived NEN tumor fragments into the CAM in an *ex ovo* setting. Traditionally, in mouse studies, successful engraftment is defined by the ability of xenografts to grow in size. In this study, we did not observe a noticeable increase in xenograft size (data not shown). We speculate that this was mainly because the patient samples used in this study had a low proliferation rate, with Ki-67 < 10% (except OCC9). Other intrinsic factors related to the biology of neuroendocrine tumors might also be responsible, such as the composition of the extracellular matrix and the presence of other cells of the tumor microenvironment that suppress the growth of neuroendocrine cancer cells. In contrast, we observed a marked increase in the tumor volume in xenografts derived from human cell lines of high-grade neuroendocrine neoplasms, suggesting that the avian model provides the right environment and nutrients for neuroendocrine cancer cells to grow. Future studies examining the role of the tumor microenvironment and characterizing the establishment of xenografts derived from tumor fragments of high-grade neuroendocrine neoplasms, such as G3-NETs and neuroendocrine carcinomas, in the avian model are warranted.

In this study, successful engraftment was confirmed by bright-field imaging, ultrasound-based blood flow assessment, and histological characterization of tumors, demonstrating that the xenografts remained viable throughout the assay. We demonstrated high engraftment rates for NET fragments across different tissue origins, tumor grades, and patient sexes. We showed that the NET PDXs retain the histopathological characteristics of the original patient samples, including tumor nest architecture and proliferation rate. The low proliferation rate, as indicated by Ki-67, is characteristic of low-grade NETs and was maintained in the PDXs. The latter suggests that there were likely no genomic alterations that contributed to a change in tumor grade; however, future studies are needed to examine this further. NET PDXs also retained SSTR2 expression as observed in the corresponding patient samples. Given the clinical significance of targeting the SSTR2 receptor with radionuclides, the NET PDXs in the avian embryo model could aid in understanding the pathways that modulate SSTR2 expression and in identifying new therapies to enhance the efficacy of radioligand therapy (48, 49, 50, 51). Finally, we demonstrated that PDXs in the avian model are vascularized, and the *ex ovo* format of this model allows imaging and quantification of both tumor vascularity using ultrasound. This can provide an additional advantage over other methods, such as organoids, for assessing the effectiveness of drugs and therapeutic approaches targeting angiogenesis in NETs, including antiangiogenic therapies (29, 52, 53, 54).

Tumor fragments from neuroendocrine liver metastasis have recently been used to generate precision-cut tumor slices (PCTSs) (45), while intact patient-derived tumor fragments (PDTFs) from various types of human cancers have been used *ex vivo* to study responses to immune checkpoint inhibitors (55, 56, 57). The *ex vivo* PCTS and PDTF models have durations comparable to those of the avian model reported here and aim to preserve the original tumor clinicopathological characteristics, thereby providing a reliable preclinical model for personalized medicine. Although *ex vivo* PCTS and PDTF models do not require a host, the avian model has a functional vascular network that can be used to address specific questions, such as those related to antiangiogenic therapy and drug delivery. In the case of the PDTF model, it was shown that patient immune cells remain viable for up to 48 h, making it suitable for dissecting responses to immune checkpoint inhibitors. Whether the immune compartment remains viable and for how long in the avian model is an active area of research within our groups. Additional studies are required to understand the specific strengths and limitations, feasibility, and reproducibility of each model for neuroendocrine cancers.

We anticipate that our platform for generating NET PDX models could be informative for examining specific biological questions, such as responses to treatments and the identification of intrinsic resistance mechanisms. As discussed above, we did not observe an increase in tumor volume in the PDX models established from low-grade patient-derived NET samples. We anticipate that PDX tumor shrinkage or other parameters, such as markers of apoptosis (e.g. cleaved caspase 3) and the activation status of specific pathways (e.g. phosphorylation of tyrosine kinase receptors), would be more appropriate for quantifying treatment response for PDX models derived from low-grade NETs in the avian model. Our PDX platform could also be used to study responses to new biological therapies, such as CAR-T cell therapies targeting specific NET antigens and antibody–drug conjugates (33, 58). On the other hand, given the relatively short duration of the assay, it is unlikely to be informative to examine mechanisms of acquired resistance after prolonged treatment. As the avian embryo serves as the host in this model, its applicability to examining the toxicity of NET therapies in adult patients is also limited. There are additional open questions regarding the utility of the PDXs in avian embryos, such as whether they are amenable to genetic manipulation, including CRISPR-mediated gene inactivation, and whether they could be used to study interactions between cancer cells and cells of the tumor microenvironment.

Overall, our study demonstrated the feasibility of establishing preclinical models, PDXs, in the avian *ex ovo* system from low-grade, well-differentiated NETs. This model could easily be adopted in research laboratories and used to dissect the biology and heterogeneity of NETs. In addition, we anticipate that PDXs generated in the avian embryos could inform physicians about the most effective treatment options for patients at specific stages of disease progression.

### Study limitations

One caveat of our study is that we had only two samples from one patient with PanNET, as surgical resection is not always indicated for this type of NETs in our center. However, given the similar engraftment rates to SI-NETs, we believe that the avian *ex ovo* model is suitable for PanNET tissue samples. Another caveat is that we had only one sample from high-grade G3 NETs. Nevertheless, given our high engraftment rate with low-grade G1/G2 NETs, the avian *ex ovo* model is likely suitable for high-grade NETs and NECs. Finally, in this study, we did not perform any genomic and transcriptomic characterization of the PDXs. While the aforementioned methods have been used to characterize other preclinical models, their utility may vary depending on the specific scientific question being addressed.

## Supplementary materials



















## Declaration of interest

The authors declare that there is no conflict of interest that could be perceived as prejudicing the impartiality of the work reported.

## Funding

This study was funded by a Pilot Award from NETRF to IPM and HSL, and a Terry Fox New Frontiers Program Project Grant to HSL and IPM (TFRI project #1124). Research in the IPM laboratory was supported by the Canadian Foundation for Innovation John R Evans Leaders Fund grant and CIHR project grant (497392). NK was supported by a fellowship from the Canadian Cancer Society and the Terry Fox Research Institute. IPM was supported by a Tier 2 Canadian Research Chair.

## Author contribution statement

NK, HSL, and IPM conceived and designed the study. NK, RC, YL, EC, ZW, SM, ALP, HSL, and IPM were involved in the development and methodology. NK, RC, and ZW acquired the data. NK, RC, EC, ZW, LC, WYH, HSL, and IPM analyzed and interpreted the data. TN, AM, PHE, and JS provided resources and critical material. RGC, HT, CL, JH, SS, CED, HSL, and IPM supervised the study. HSL and IPM coordinated the study. NK, HSL, and IPM wrote the manuscript. All authors reviewed and revised the manuscript and approved the final version of the manuscript
