# Supplementary figures and images for: Establishment of patient-derived xenografts for neuroendocrine tumors in the avian embryo model
Source: Endocr Relat Cancer. 2026 Mar 23;33(3):e250377. doi: 10.1530/ERC-25-0377 (PMC13034492; doi:10.1530/ERC-25-0377)

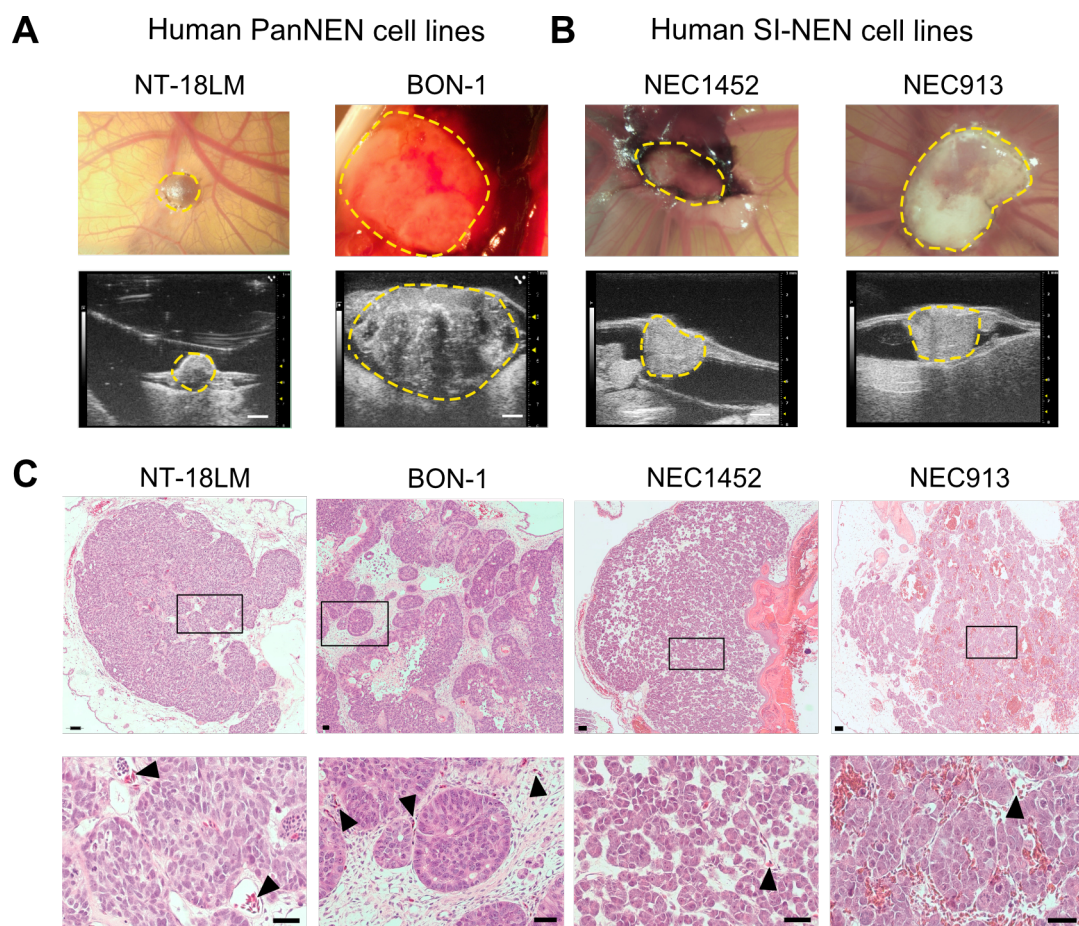

**Supplementary Figure 1: Tumor establishment from human NEN cell lines within the avian embryo.**

Supplement: Supplementary file 1 [file supplementary_figure_1.pdf]

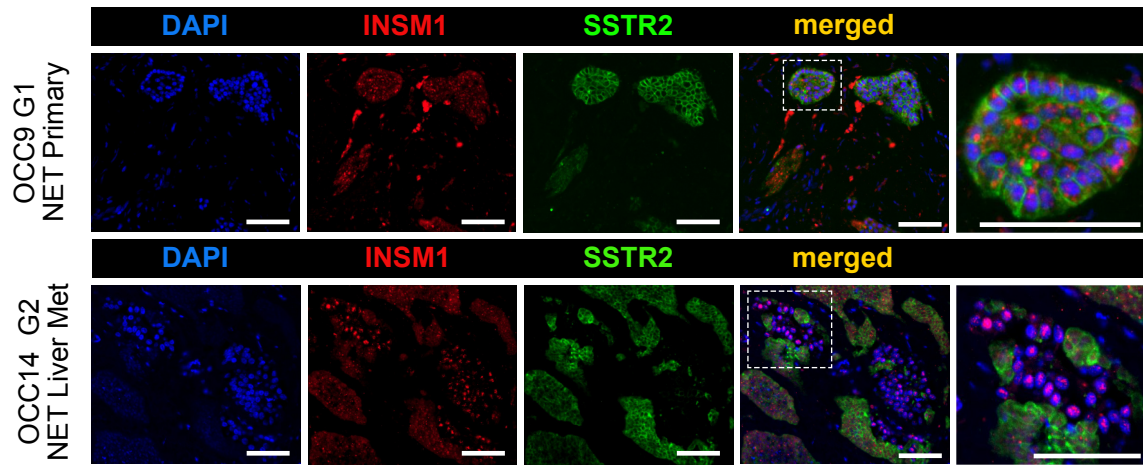

**Supplementary Figure 5: Somatostatin-receptor 2 (SSTR2) expression in human-derived NET PDXs.**

Supplement: Supplementary file 5 [file supplementary_figure_5.pdf]
