# Supplementary material for: Establishment of patient-derived xenografts for neuroendocrine tumors in the avian embryo model
Source: Endocr Relat Cancer. 2026 Mar 23;33(3):e250377. doi: 10.1530/ERC-25-0377 (PMC13034492; doi:10.1530/ERC-25-0377)
Supplement: Supplementary file 2 [file supplementary_figure_2.pdf]

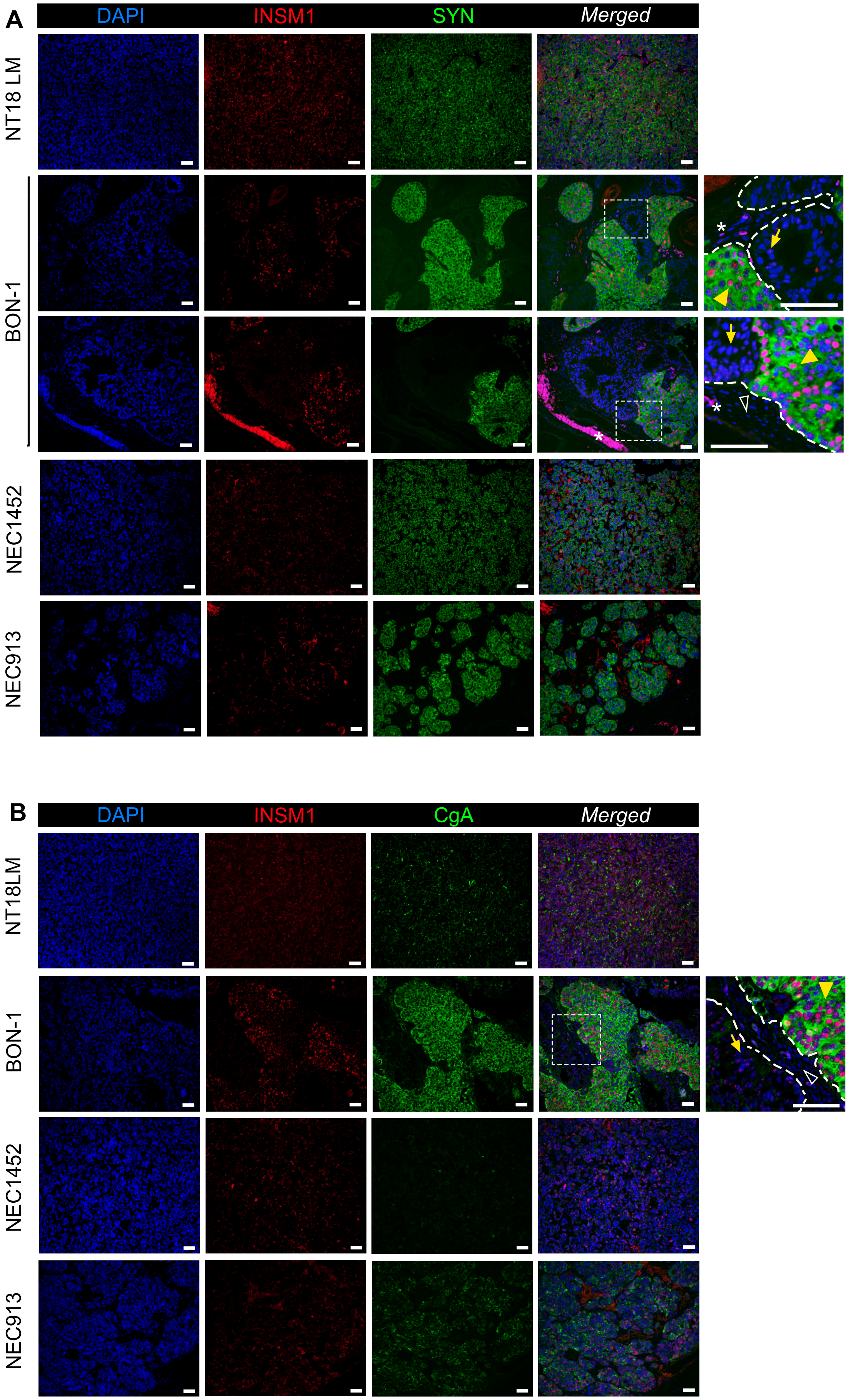

Supplementary Figure 2: Expression of neuroendocrine markers in tumors established from human NEN cell lines within the avian embryo.
